# Association of *qnr* Genes and OqxAB Efflux Pump in Fluoroquinolone-Resistant *Klebsiella pneumoniae* Strains

**DOI:** 10.1155/2023/9199108

**Published:** 2023-02-21

**Authors:** Fereshteh Amereh, Mohammad Reza Arabestani, Seyed Mostafa Hosseini, Leili Shokoohizadeh

**Affiliations:** ^1^Student Research Committee, Hamadan University of Medical Sciences, Hamadan, Iran; ^2^Infectious Disease Research Center, Hamadan University of Medical Sciences, Hamadan, Iran; ^3^Department of Microbiology, Faculty of Medicine, Hamadan University of Medical Sciences, Hamadan, Iran

## Abstract

**Background:**

The aim of this study was to investigate the frequency and relationship between plasmid-mediated quinolone resistance genes and OqxAB pump genes, as well as the genetic linkage in *K. pneumoniae* strains isolated from Hamadan hospitals in the west of Iran.

**Materials and Methods:**

In this study, 100 *K. pneumoniae* clinical strains were isolated from clinical samples of inpatients at Hamadan Hospital in 2021. The antimicrobial susceptibility testing was performed using the disk diffusion method. The frequencies of genes encoding OqxAB efflux pumps and *qnr* were investigated by PCR. Molecular typing of *qnr*-positive *K. pneumoniae* isolates was assessed by ERIC-PCR.

**Results:**

Antibiotic susceptibility testing showed high resistance (>80%) to fluoroquinolones. The gene encoding the OqxAB efflux pump was detected in more than 90% of *K. pneumomiae* strains. All *K. pneumoniae* isolates were negative for *qnr*A, and 20% and 9% of the isolates were positive for *qnr*B and *qnr*S, respectively. The genes encoding *oqx*A and *oqx*B were detected in 96% of *qnr*-positive strains. A *qnr*B + /*qnr*S + profile was observed in 16% of *qnr*-positive *K. pneumoniae* strains. Ciprofloxacin MIC ≥ 256 *μ*g/ml was detected in 20% of *qnr*-positive strains. Genetic association analysis by ERIC-PCR revealed genetic diversity among 25 different *qnr*-positive strains of *K. pneumonia*.

**Conclusion:**

However, no significant correlation was found between the *qnr* and the OqxAB efflux pump genes in this study. The high rate of fluoroquinolone resistance and determinants of antibiotic resistance among diverse *K. pneumoniae* strains increase the risk of fluoroquinolone-resistance transmission by *K. pneumoniae* strains in hospitals.

## 1. Introduction

One of the causes of pneumonia, sepsis, and urinary tract infections in hospitalized patients is known to be *Klebsiella pneumoniae*, a Gram-negative bacterium in the Enterobacteriaceae family. This bacterium is more likely to infect people whose immune systems are weak. The majority of antibiotics used to treat infections caused by this bacterium are beta-lactam and fluoroquinolones [1].

Controlling antibiotic resistance in multidrug-resistant *K. pneumoniae* (MDR-KP) is a major challenge. Optimal treatment options for MDR-KP infections are not yet well established. New antimicrobial agents against MDR-KP have been developed over the past decades and are currently in various stages of clinical research [2]. Fluoroquinolones such as ciprofloxacin are among the antibiotics used to treat infections associated with *K. pneumoniae* [3]. Quinolones and fluoroquinolones represent a wide range of antibiotics whose mode of action is to inhibit bacterial DNA gyrase enzymes [4]. In recent years, increasing resistance to these antibiotics has led not only to problems in the treatment procedure but also to increased treatment costs and longer hospital stays. Therefore, there is a need to assess resistance levels and mechanisms of resistance to these antibiotics in nosocomial pathogens such as *K. pneumonia* infections [5].

Resistance to ciprofloxacin can develop through a variety of mechanisms. Ciprofloxacin resistance is primarily associated with chromosomal mutations that alter ciprofloxacin target proteins (DNA gyrase and topoisomerase IV). Quinolone resistance can also be induced by mutations in the Efflux pump gene regulators. In addition to mutations in susceptible targets, ciprofloxacin resistance can also be mediated by plasmid-mediated quinolone resistance (PMQR) genes and efflux pumps such as OqxAB [4]. The OqxAB is expressed on plasmids in clinical isolates of *E. coli* and *K. pneumoniae* [6]. The qnr plasmid protects ciprofloxacin targets from inhibition and causes low-level resistance to quinolone. Different *qnr* genes have been found in bacterial strains from various regions of the world. Many qnr genes have been discovered including *qnr*A, *qnr*S, *qnr*B, *qnr*C, and *qnr*D and more recently *qnr*VC and *qnr*T [6, 7].

The aim of this study was to investigate the frequency and relationship between plasmid-mediated quinolone resistance (*qnr*) genes and OqxAB pump genes (*oqx*A and *oqx*B), as well as the genetic linkage in *K. pneumoniae* strains isolated from Hamadan hospitals in the west of Iran.

## 2. Methods

### 2.1. Bacterial Isolation and Identification

A total of 100 clinical isolates of *K. pneumoniae* were randomly collected in 2021 from patients at three major hospitals in Hamadan. *K. pneumoniae* isolates were identified by routine microbiological testing. Lactose-fermenting mucoid (pink-colored) colonies on MacConkey agar were selected. *K. pneumoniae* isolates were identified by IMVIC test (Indole production, Methyl Red (MR), Voges Proskauer (VP), and Simmons citrate), as well as by SIM, TSI, Urea Hydrolysis, and Lysine Decarboxylase tests. The results of the biochemical assay were confirmed by PCR using species-specific primers for the *ure*D gene responsible for urea hydrolysis in *K. pneumoniae* [8, 9].

### 2.2. Antimicrobial Susceptibility Testing

Antibiotic susceptibility testing of *K. pneumoniae* strains was performed according to CLSI criteria [10]. Susceptibility to piperacillin (100 *μ*g), chloramphenicol (30 *μ*g), nitrofurantoin (300 *μ*g), imipenem (10 *μ*g), gentamicin (10 *μ*g), amikacin (30 *μ*g), ceftazidime (30 *μ*g), ceftriaxone (30 *μ*g), cefotaxime (30 *μ*g), ciprofloxacin (5 *μ*g), levofloxacin (5 *μ*g), and ampicillin-sulbactam (10/10 *μ*g) were detected by the disk diffusion method, and the minimum inhibitory concentration (MIC) of ciprofloxacin (Sigma, Germany) was determined by the broth microdilution method. *E. coli* ATCC 25922 was used as a quality control strain.

### 2.3. Molecular Detection of *qnr* and Efflux Pump Genes

Genomic DNAs from ciprofloxacin-resistant *K. pneumonia*e isolates was extracted using the boiling method [11]. The presence of *qnr*A, *qnr*B, *qnr*S, *oqx*A, and *oqx*B genes was investigated by PCR using specific primers as previously described [12, 13]. The PCR reaction mixture contained 3 *μ*l template DNA, 12.5 *μ*l 2X Taq premix (Ampliqune Co, Denmark), 2 *μ*l each primer (Metabion Co, Germany), and 3.5 *μ*l ddH2O in a final volume of 25 *μ*l. The PCR procedure OqxAB efflux pump genes and of *qnr*A, *qnr*B, and *qnr*S were performed in a thermal cycler (Bio-Rad, Inc. USA), according to the program shown in Table 1. Detection of PCR products was performed with a 100 bp DNA ladder on a 1% agarose gel. The sizes of amplified fragments for *qnrA*, *qnrB*, *qnrS, oqxA,* and *oqxB* were 571 bp, 594 bp, 388 bp, 207 bp, and 512 bp, respectively [12, 13].

### 2.4. ERIC-PCR

All *qnr*-positive *K. pneumoniae* strains were selected for molecular typing by ERIC-PCR. This procedure was performed using the primers as described previously [12] and the program in Table 1. The ERIC profiles were analyzed by an online database analysis service (insilico.ehu.es). A cutoff similarity of ≥95% was considered.

### 2.5. Data Analysis

Data were analyzed using SPSS software version 22.0 (IBM Co., Armonk, NY, USA). The relationships between different *qnr* genes, OqxAB efflux pump genes, antibiotic resistance, sample source, and hospital wards were analyzed using the Pearson chi-squared or fisher exact test. The relationship between *qnr* genes and the ciprofloxacin MIC values was analyzed by the *t*-test. *P* values less than 0.05 were considered statically significant.

## 3. Results

### 3.1. Phenotypic Characteristics of *K. pneumoniae* Isolates

*Klebsiella pneumoniae* colonies were observed as pink mucoid colonies on McConkey agar. According to biochemical tests, *K. pneumoniae* colonies were indole and MR negative, and VP, Simmons citrate, urea hydrolysis, and lysine decarboxylase tests were positive. The presence of the *ure*D gene in *K. pneumonia* isolates were confirmed by PCR.

### 3.2. Antimicrobial Susceptibility Testing

Disk diffusion results showed a high level of resistance (89%) to ciprofloxacin. According to the CLSI 2021 criteria, ciprofloxacin breakpoints of ≥0.25 *μ*g/ml, 0.5 *μ*g/ml, and ≥1 *μ*g/ml were considered susceptible, intermediate, and resistant strains. In this study, the MIC values of ciprofloxacin ranged from ≥0.5 *μ*g/ml to ≥256 *μ*g/ml. Ciprofloxacin MIC ≥ 256 *μ*g/ml was detected in 26% and 20% of ciprofloxacin-resistant and *qnr*-positive strains, respectively. A multidrug resistance (MDR) phenotype was found in 96% (24 isolates) of the *qnr*-positive strains (Table 2). The non-MDR strain with a ciprofloxacin MIC > 2 *μ*g/ml was negative for the efflux pump genes (Table 2).

### 3.3. Prevalence of *qnr* and Efflux Pump Genes

**Figure 1:**
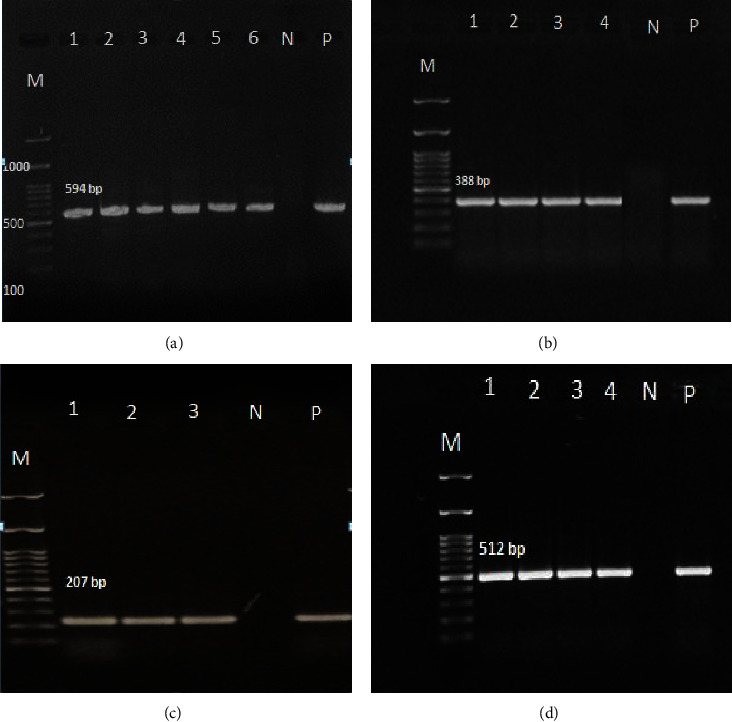
Gel electrophoresis of PCR products of (a) *qnr*B (594bp), (b) *qnr*S (388bp), (c) *oqx*A (207bp), and (d) *oqx*B (512bp) in qnr-positive *Klebsiella pneumoniae* strains isolated from hospitalized patients in Hamadan hospitals. Lane M: 100 bp DNA size marker, lane P: positive control, and lane N: negative control.

**Figure 2:**
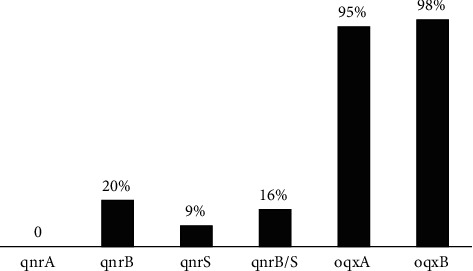
The prevalence of qnr and efflux pump genes is *K. pneumoniae* strains.

The results of *qnr-*genes detection by PCR showed that all *K. pneumoniae* isolates were negative for *qnr*A, and 20% and 9% of the isolates were positive for *qnr*B and *qnr*S, respectively (Figure 1). A *qnr*B+/*qnr*S + profile was observed in 16% of *qnr*-positive *K. pneumomiae* strains. The *oqx*A and *oqx*B genes were detected at 95% and 98% of *K. pneumoniae* isolates, respectively (Figure 2). According to the results, the gene encoding *oqx*A and *oqx*B were detected in 24 (96%) of *qnr-*positive strains. Table 2 provides information on isolates containing the *qnr* genes. Static analysis of the results showed that there were no significant correlations between *qnr* genes, efflux pumps, ciprofloxacin resistance, hospital wards, sample sources, ciprofloxacin MICs, or patterns of antibiotic resistance (*P* value ≥0.05). However, efflux pump genes and ciprofloxacin resistance were found to be significantly correlated (*P* value ≤0.04).

**Figure 3:**
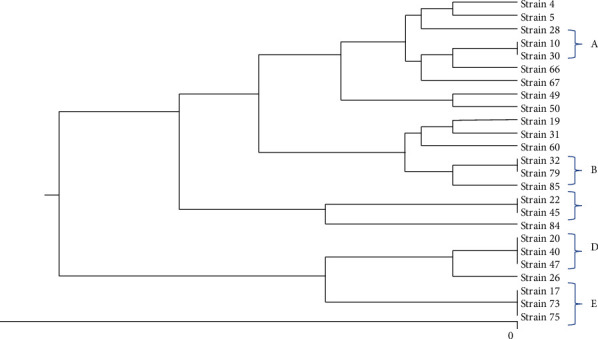
Dendrogram of ERIC-PCR types of qnr-positive *K. pneumoniae* strains, compared by dice method and grouped by UPGMA method.

ERIC-PCR analysis of genetic linkage revealed genetic diversity among 25 different *K. pneumonia qnr*-positive strains. The results of the ERIC-PCR revealed the presence of 18 different ERIC profiles, including 5 common types and 13 unique types (including one isolate). There were two to three isolates in the common types (Figure 3). Our findings revealed the distribution of MDR and fluoroquinolone-resistant *K. pneumoniae* strains in some Hamadan hospital wards, as well as the distribution of related *K. pneumoniae* clones (ERIC type) in some hospital wards (ERIC types A, B, and E in the intensive care units). Strains of a common type showed different patterns of antibiotic resistance and different profiles of efflux pumps and *qnr* genes, and no significant relationship was observed between them (Table 2). There were no significant correlations between *qnr* genes and ERIC types.

## 4. Discussion

Fluoroquinolone resistance is an important problem associated with *K. pneumoniae*. Fluoroquinolone resistance has been suggested to play an important role in the successful evolution of *K. pneumoniae* strains [14]. This study showed a high level (≥80%) of resistance to ciprofloxacin in *K. pneumoniae* strains. Most fluoroquinolone-resistant strains also display the MDR phenotype. This finding highlights the problem of management of *K. pneumoniae* infections in hospitals and poses significant challenges for clinicians. Many of the isolates in this study were isolated from patients admitted to the ICU, which may be one of the main reasons for the high resistance to ciprofloxacin in this study. The rate of fluoroquinolone resistance in our study is higher than that in the study in Iran and some other regions [15–20]. However, a recent study of Hamadan reported a high rate of resistance (≥80%) to fluoroquinolones [21]. In total, our findings indicate that the *qnr* genes were present in 25% of *K. pneumoniae* isolates. None of the isolates contained *qnr*A, and *qnr*B was identified as the most prevalent *qnr* gene. The prevalence of *qnr*B and *qnr*S was 20% and 9%, respectively, in the isolates. On the distribution of the *qnr* gene in *K. pneumoniae*, there are various reports, including those from Iran. Nourozi et al. reported that *qnr*B (43% of isolates) was the most frequently detected *qnr* gene, followed by *qnr*S (34% of isolates) and *qnr*A (23% of isolates) in *K. pneumoniae* isolates from hospitals in Tehran [22]. The study results of Salimbahrami et al. showed that 47 (52%), 22 (25%), and 21 (23%) *K. pneumoniae* isolated from hospitals in Sari (northern Iran) contains the *qnr*B, *qnr*A, and *qnr*S genes, respectively [16]. In another study from southwestern of Iran by jomehzadeh et al., ciprofloxacin resistance was lower than our results while *qnr*A, *qnr*B, and *qnr*S were detected in (12%), (24%), and 17% of *K. pneumonia* isolates [23]. In the following, studies that reported results similar to ours are reviewed. In a study conducted by Malek Jamshidi et al., at Yazd central laboratory, the *qnr*A gene was not detected in any of the *K. pneumoniae* strains, and the *qnr*B gene (25%) was identified as dominant [23]. In a study from India, 22% of isolates possessed both *qnr*B and *qnr*S genes, while the *qnr*A gene was not detected in any strains [19]. Studies in China, Singapore, and Malaysia have demonstrated that *qnr*B is the major *qnr* gene, which is consistent with our findings and the majority of Iranian studies [24–26].

The differences in *qnr* genes frequency distribution of the of *K. pneumoniae* strains in different studies may be due to differences in geographical distribution of fluoroquinolone-resistant strains, sampling locations, and infection control strategies in the hospitals. Most *qnr*-positive strains had increased MICs for ciprofloxacin (28% and 20% of *qnr*-positive strains, MIC ≥ 128, and MIC  ≥ 256, respectively). These results indicate that the presence of the *qnr* gene may play a role in reducing susceptibility to fluoroquinolones. It has also been shown that the *qnr* genes products can protect fluoroquinolone targets from antibiotic action. These genes are widely distributed in Enterobacteriaceae. The *qnr* gene is thought to induce low to moderate resistance to quinolones. Factors other than PMQR, such as *gyr*A and *gyr*B mutations and efflux pumps, may also play a role in the emergence of fluoroquinolone resistance. Plasmid-mediated *qnr* gene transfer between nosocomial pathogens increases the risk of transmitting resistance to fluoroquinolones and reduces the susceptibility of these pathogens to these antibiotics.

Another important factor in resistance to fluoroquinolones is the efflux pump. In this study, we investigated the association of the OqxAB efflux pump with resistance to fluoroquinolones. In this study, >95% of the isolates carried the OqxAB efflux pump genes. The OqxAB gene has been repeatedly reported in quinolone-resistant Enterobacteriaceae [27]. Expression of OqxAB has been shown to be associated with reduced susceptibility to quinolones in *K. pneumonia* [28, 29]. The present study found a significant relationship between ciprofloxacin resistance and the presence of OqxAB efflux pumps. The percentage of the OqxAB genes in our study is higher than that in other Iranian studies [22, 30, 31]. In our study, 96% of the *qnr*-positive strains harbored the efflux pump genes and exhibited the MDR phenotype. High ciprofloxacin resistance may be related to the coexistence of the efflux pump OqxAB and the *qnr* genes.

For molecular typing of *K. pneumoniae* isolates, our study used ERIC-PCR, which is a simple, inexpensive, and accessible method with sufficient reliability and reproducibility. Such as many studies, genetic diversity has been distinguished among *K. pneumoniae* strains [12, 32–34]. Our results demonstrated the prevalence of related *K. pneumoniae* strains (ERIC type) in some hospital wards, as well as the distribution of MDR and fluoroquinolone-resistant *K. pneumoniae* strains in some hospital wards, suggesting that these strains need further investigation. In this study, antibiotic resistance profiles and *qnr* gene profiles of strains belonging to the same ERIC class were different. To learn more about resistant strains, we recommend further investigation of the different mechanisms that influence the genetic differentiation of *K. pneumoniae*. OqxAB efflux pump genes and antibiotic resistance patterns were not significantly correlated with the ERIC groups, as shown in our results. Our results also show that widespread *K. pneumoniae* colons with different antibiotic resistance patterns and high proportions of antibiotic resistance determinants such as oqxAB and *qnr* genes can interfere with the control of nosocomial infections. In addition, high-risk fluoroquinolone-resistant *K. pneumoniae* clones have been shown to maintain fitness, facilitating their spread in hospital settings [35].

## 5. Conclusion

However, the results of this study suggested that there is no significance association between PMQR and OqxAB efflux pump genes but the alarming prevalence of MDR phenotypes, presence of OqxAB efflux pump, and PMQR determinants in heterogeneous fluoroquinolone-resistant *K. pneumoniae* strains in hospitals increase the risk of fluoroquinolone-resistance transmission among hospital-adapted pathogens and cause challenges for clinicians in hospitals. More bacterial strains should be tested and a wider area sampled for more accurate and better results. All of this requires research funding and more collaboration between hospitals, research centers, and universities.

## Figures and Tables

**Table 1:** PCR program for detection of *oqx*A and *oqx*B and *qnr* genes.

Primer	Initial denaturation	Denaturation	Annealing	Elongation	Final extension	Cycle
*OqxA*	94°C	94°C	56°C	72°C	72°C	25
	2 min	15 s	30 s	1 min	7 min	

*OqxB*	94°C	94°C	55°C	72°C	72°C	32
	2 min	30 s	30 s	1 min	10 min	

*qnrA*, *B*, *S*	94°C	94°C	57°C	72°C	72°C	30
	5 min	1 min	1 min	1 min	5 min	

**Table 2:** Characteristics of qnr-positive *K. pneumoniae* strains based on source, hospital wards, antibiotic resistance, *qnr*s and oqxAB genes profiles, and ERIC types.

Isolate	Source	Ward	CIP MIC	*oqx*A	*oqx*B	*qnr*B	*qnr*S	ERIC type	Antibiotic resistance patterns
4	Urine	Internal	32	+	+	+	−	Single	PIP, CL, NIT, IPM, and LEV
5	Trachea	ICU	128	+	+	+	−	Single	PIP, CTX, GM, SAM, NIT, CIP, and LEV
10	Blood	ICU	128	+	+	−	+	A	CTX, GM, SAM, CIP, and LEV
17	Wound	Internal	128	+	+	+	−	E	PIP, CAZ, CRO, IMP, AMK, GM, CIP, and LEV
19	Urine	ICU	64	+	+	+	−	Single	PIP, IPM, CL, GM, and NIT
20	Trachea	ICU	2	−	−	+	−	D	SAM, CAZ, and CIP
22	Urine	Emergency	64	+	+	+	−	C	PIP, CTX, CRO, IPM, CL, LEV, and CIP
26	Urine	Internal	128	+	+	+	−	Single	PIP, CTX, SAM, IPM, AMK, CL, GM, CIP, and LEV
28	Urine	ICU	128	+	+	+	−	Single	SAM, CL, NIT, GM, CIP, and LEV
30	Blood	ICU	256	+	+	−	+	A	SAM, CL, GM, CIP, and LEV
31	Trachea	ICU	256	+	+	+	−	Single	CRO, CTX, SAM, CL, NIT, LEV, and CIP
32	Urine	ICU	32	+	+	+	+	B	GM, NIT, and CIP
40	Sputum	ICU	256	+	+	+	−	D	PIP, CAZ, CRO, CTX, IMP, AMK, GM, CIP, and LEV
45	Urine	ICU	128	+	+	+	−	C	CRO, CTX, SAM, AMK, GM, CL, IMP, CIP, and LEV
47	Urine	Internal	128	+	+	−	+	D	CRO, CTX, SAM, AMK, GM, CL, NIT, CIP, and LEV
49	Trachea	Internal	32	+	+	+	−	Single	PIP, CAZ, CRO, CTX, SAM, AMK, GM, CIP, and LEV
50	Sputum	ICU	64	+	+	+	+	Single	CAZ, CRO, CTX, SAM, IPM, GM, NIT, CIP, and LEV
60	Plural	Internal	32	+	+	+	−	Single	PIP, CRO, SAM, NIT, CIP, and LEV
66	Urine	ICU	8	+	+	+	−	Single	CAZ, CRO, CTX, SAM, AMK, GM, NIT, CIP, and LEV
67	Urine	ICU	32	+	+	+	+	Single	PIP, CAZ, SAM, GM, CIP, and LEV
73	Trachea	ICU	256	+	+	+	−	E	PIP, CAZ, CRO, CTX, SAM, CL, NIT, AMK, GM, CIP, and LEV
75	Urine	ICU	32	+	+	−	+	E	CRO, CTX, SAM, CL, NIT, AMK, GM, IMP, CIP, and LEV
79	Wound	ICU	16	+	+	−	+	B	PIP, CTX, SAM, IMP, NIT, CIP, and LEV
84	Trachea	Internal	256	+	+	+	−	Single	PIP, CRO, CAZ, CTX, SAM, IMP, NIT, CIP, and LEV
85	Trachea	Internal	64	+	+	+	+	Single	PIP, CAZ, SAM, CL, AMK, GM, IMP, NIT, CIP, and LEV

PIP: piperacillin, CL: chloramphenicol, NIT: nitrofurantoin, IPM: imipenem, GM: gentamicin, AMK: amikacin, CAZ: ceftazidime, CRO: ceftriaxone, CTX: cefotaxime, CIP: ciprofloxacin, LEV: levofloxacin, and SAM: ampicillin-sulbactam.

## Data Availability

The data used to support the findings of this study are included in the article.
